# Cross-sectional study for derivation of a cut-off value for identification of an early versus delayed diagnosis of endometriosis based on analytical and descriptive research methods

**DOI:** 10.1186/s12905-022-02044-x

**Published:** 2022-12-14

**Authors:** Iris Brandes, Katja Kleine-Budde, Nicole Heinze, Sebastian Binder, Constanze Klug, Cordula Schippert, Andreas D. Ebert, Gülden Halis

**Affiliations:** 1 Department of Epidemiology, Social Medicine and Health System Research, Hannover Medical School (MHH), Hannover, Germany; 2 Clinical Cancer Registry Lower Saxony (KKN), Oldenburg, Germany; 3 German Rheumatism Research Centre Berlin (DRFZ), Berlin, Germany; 4 Institute of Medical Management and Health Sciences (IMG), University of Bayreuth, Bayreuth, Germany; 5 Department of Obstetrics and Gynecology, Hannover Medical School (MHH), Hannover, Germany; 6 Gynecology & Obstetrics Berlin, Berlin, Germany; 7 Fertility Team Berlin, Berlin, Germany

**Keywords:** Endometriosis, Diagnostic delay, Cut-off value, Early onset of endometriosis-related symptoms

## Abstract

**Background:**

Endometriosis is a benign, hormone-dependent, chronic inflammatory gynecological disease accompanied by cyclic and acyclic pelvic pain and other complaints. The long lists of research recommendations in the AWMF guideline (Burghaus et al., Geburtshilfe Frauenheilkd 81:422–46, 2021) and ESHRE Endometriosis Guideline (ESHRE Endometriosis Guideline Development Group, Endometriosis: Guideline of European Society of Human Reproduction and Embryology, 2022) show that there is still a great need for research in all aspects of the disease. Diagnostic delay, defined as the mean time between symptom onset and confirmed diagnosis, is a particular problem associated with endometriosis. Some quantitative and qualitative studies have investigated possible reasons for this. A range of physician-related (Dixon et al., Br J Gen Pract 71:e668-e676, 2021; van der Zanden and Nap, Reprod Biomed Online 32:527–31, 2016) and patient-related factors (Sayer-Jones and Sherman, Health Psychol Behav Med 9:456–79, 2021) as well as stigmatization of the topic of menstruation by society have been identified (Kruckenberg, Frauenarzt 59:2–5, 2018; Seear, Soc Sci Med 69:1220–7, 2009). The consequences of the disease being diagnosed late (or too late) on the course of disease, the quality of life and the costs of the disease have already been documented in studies (Sims Int J Environ Res Public Health 18(15):8210, 2021; Surrey Adv Ther 37:1087–99, 2020).

However, a systematically derived cut-off value that clearly distinguishes between short and long delay is still lacking. Therefore, the aim of our study was to derive a threshold value for the definition of a target corridor for endometriosis diagnosis based on descriptive and analytical methods.

**Methods:**

Since our review of the rather sparse publications on diagnostic delay did not yield satisfactory results, we used descriptive statistics and location parameters to calculate a cut-off value for German population data from the EndoCost study. Statistical methods were used for correlation analysis of shortDD versus longDD (correlation analysis and logistic regression) and group membership (discriminant analysis).

**Results:**

Five years was identified as the cut-off value that significantly differentiated between shortDD and longDD based on various disease-related variables. This suggests that endometriosis should be definitively diagnosed within less than five years to minimize the risk of an unfavorable course of the disease.

**Conclusion:**

Our findings confirmed that an early onset of endometriosis-related symptoms is the most important risk factor for a long diagnostic delay. Consequently, adolescent females should receive increased attention as an especially vulnerable group. Evidently, there is an urgent need to develop adequate concepts to improve the endometriosis education and care among this target group.

## Background

Endometriosis is a chronic inflammatory disorder in which tissue similar to that which lines the inside of the uterus grows outside the uterine cavity [1, 2]. The cardinal symptoms of endometriosis are primary or secondary dysmenorrhea, chronic recurrent abdominal pain, pain during sexual intercourse, abdominal distension, dysuria and infertility [2–5]. Various studies have shown that endometriosis reduces the quality of life of affected women [6–9]. In addition to having physical, mental and social consequences for affected women at the individual level [10], endometriosis is estimated to have significant economic impacts on society as a whole [11–13], due mainly to increased healthcare utilization and reduced work productivity [12, 14, 15]. Endometriosis is estimated to affect 4–10% of women of reproductive age, making it one of the most common benign gynecological conditions [16–18].

Although the diagnostic delay of endometriosis, defined as the time from the onset of endometriosis symptoms until confirmed diagnosis, is a central problem discussed in the literature [19–22], comprehensive and differentiated studies on this topic are still scarce. In particular, a systematically derived decision limit for discriminating between a “short” and a “long” time to diagnosis of the disease is still lacking. Surrey et al. [23] divided the diagnostic delay interval into three categories: short (≤ 1 year), intermediate (1–3 years) and long (3–5 years). Their classification is intuitive but lacks methodological rigor. The identification of a cut-off point would enable more comprehensive study of the consequences of the delay in diagnosis of endometriosis. Preliminary evidence suggesting that a long delay in diagnosis of endometriosis is associated with a higher individual burden of disease, disease mismanagement, and higher healthcare costs attest to the need for further research on this topic [23], Future research should focus, in particular, on the impact of endometriosis diagnostic delay on health-related quality of life – the most important patient-related outcome [10, 14, 24, 25].

Therefore, we utilized data from German population of the EndoCost study [26, 27] to identify and empirically test a potentially suitable cut-off point as a theory-based measure of endometriosis diagnostic delay. The aim was to identify an appropriate decision limit using various statistical methods.

## Methods

### Data basis

The results of the present study are based on patient questionnaire data from the German population of the EndoCost study, a multi-center, prevalence-based bottom-up study conducted at endometriosis centers in ten countries (Germany, Denmark, Switzerland, Hungary, Belgium, the Netherlands, Italy, France, UK and USA). The aim of the study was to gather information on a wide range of disease-specific parameters, healthcare costs and health-related quality of life of endometriosis patients from a societal perspective [26, 27]. In September 2009, 788 potential participants were recruited at EndoCost centers in Germany. Inclusion criteria were a histologically confirmed diagnosis of endometriosis and utilization of at least one endometriosis-related healthcare service at one of the participating study centers (Humboldt Clinic Berlin, Berlin Fertility and Endometriosis Center, Hannover Medical School) in 2008. Written data was collected using a 30-page questionnaire including items regarding the patients’ demographic and disease-specific characteristics.

### Cut-off point selection

The results presented here are based on a differentiated analysis of women with a “short” versus “long” delay in histological diagnosis of endometriosis. Cut-off point selection was performed as a two-part process. The first consisted of a comprehensive search and review of the literature on studies reporting data on the average diagnostic delay for endometriosis. The aim was to derive a threshold value based on the available empirical data. A search was conducted of the National Library of Medicine’s MEDLINE/PubMed databases with the intent of finding all articles published in the English or German language with “endometrios*” in conjunction with “diagnostic delay”. 59 Articles published from 1997 to 2022 were identified by this search strategy. The abstracts of these articles were read and analysed for relevance. Additionally, corresponding bibliographic reference sections were reviewed for additional studies not found by the previous method. Two articles were found, so that a total of sixty (61) articles were reviewed. Reasons for exclusion were: case reports (2 articles), article not available in English or German (2 articles), no full text available (19 articles), articles reported the results of studies with qualitative study designs and did not present detailed consideration (6 articles), articles cite only data from other studies regarding the length of diagnostic delay of endometriosis. All publications in German and English that were based on an independent quantitative analysis of endometriosis diagnostic delay were included in the review (*n* = 13).

The second step involved characterization of diagnostic delay times observed in the German EndoCost study population in terms of descriptive location parameters, such as the arithmetic mean, median, minimum and maximum. For a more differentiated analysis, we divided the pre-diagnostic period into three time segments: 1) the patient delay interval (DDpatient), defined as the mean time from first symptom onset to first consultation with a physician, 2) the physician delay interval (DDphysician), defined as the mean time from first consultation to confirmed diagnosis (physician-related delay, DDphysician), and 3) the total diagnostic delay (DDtotal), defined as the mean time from first symptom onset to confirmed diagnosis.

Statistical analysis was performed using IBM SPSS Statistics Version 27 software (Statistical Package für Social Sciences) for descriptive and analytical statistics. In descriptive statistics, the mean and median were used to measure the central tendency of the data, and the standard deviation (SD) was used describe the spread of the data from the mean.

### Statistics

Differences between women with long and short diagnostic delays were characterized by comparing the means of different classes of variables, including ordinal variables, at minimum. Because the distributions were skewed (i.e., non-normal), the Mann–Whitney U test was used to determine whether the difference in means was statistically significant. Correlation analysis and logistic regression of the target variable (shortDD versus longDD) were used to determine the strength of correlation between individual patient characteristics and diagnostic delay times. Discriminant analysis was used to predict group membership (shortDD versus longDD), and collinearity values from correlation analysis and logistic regression were used to test for multicollinearity.

## Results

### Literature review

The results of the review of the literature on international quantitative studies of endometriosis diagnostic delay are summarized in chronological order of publication in Table 1. Original publications stating the time from first symptom onset to confirmation of the diagnosis of endometriosis were included in the review. In all of these studies, data was collected using retrospective questionnaires or interviews. However, the participants were recruited in different settings, including endometriosis self-help groups, an endometriosis association, and inpatient treatment centers [28]. At the time of recruitment, the latter participants were either admitted to a specialist clinic [20, 21], receiving outpatient treatment [29], or scheduled for first-time laparoscopy for endometriosis [30].

**Table 1:** International study results on diagnostic delay in endometriosis

Study	Material and Methods	Study Participants	Diagnostic delay in years^a^
Headfield et al. 1996 [31]	Retrospective questionnaire administered to women in the U.S. and U.K. (*n* = 218)	Women in previous inpatient-treatment and women of a self-help group with histologically confirmed endometriosis	USA (mean value): 11,7 ± 9,1 (SD) UK (mean value): 8,0 ± 7,9 (SD)
Arruda et al. 2003 [29]	Unicenter study; retrospective questionnaire-based interviews with women in Brasil in 2000 to 2001 (*n* = 200)	Former patients of an outpatient clinic with histologically confirmed endometriosis	Brasil (mean value): 7,0 (Range 3,5–12)
Husby et al. 2003 [28]	Retrospective questionnaire administered to women in Norway in 2001(*n* = 261)	Former patients of an inpatient clinic and members of a self-help group	Norway (mean value): 6,7 ± 6,2 (SD)
Ballard et al. 2006 [20]	Unicenter study; retrospective semi structured face-to-face interviews with women in England in 2004 to 2005 (*n* = 28)	Women referred to a hospital pelvic pain clinic	England (median): 8,5 (range 1–27)
Noaham et al. 2011 [30]	Multicentric study in 10 countries^b^ with retrospective questionnaire in 2008 to 2010 (*n* = 745)	Women scheduled for a laparosopy	10 Countries^b^: (mean value) 6,7 ± 6,3 (SD)
Hudelist et al. 2012 [21]	Multicentric study with retrospective questionnaire administered to women in Germany and Austria in 2010 to 2012 (*n* = 171)	Women with histologically confirmed endometriosis in specialized treatment centers	Germany and Austria: (mean value) 10,4 ± 7,9 (SD)
Staal et al. 2016 [32]	Unicenter study; retrospective telephone survey (questionnaire) with women in Netherlands in 2012 to 2014 (*n* = 139)	Former patients with histologically confirmed endometriosis in a specialized treatment center	Netherlands (mean value) 7,4 (range) 2,1 – 14,1
Amour et al. 2020 [33]	Cross-sectional study in Australia; online survey (retrospective questionnaire) in 2017 (*n* = 340)	Women with histologically confirmed endometriosis, recruited nationwide via media of self-help groups	Australia (mean value) 7,8 ± 10,1 (SD)
Singh et al. 2020 [34]	Cross-sectional study in Canada; online survey by using three independent response panels in 2018/2019 (*n* = 2004)	Women with self reported endometriosis, nationwide	Canada (mean value) 5,4
Surrey et al. 2020 [23]	Retrospective study of insured data in the U.S. from 1999 to 2017 (*n* = 11.793)	Women with at least one billed service for endometriosis between 2004 and 2016	USA (mean value) 2,1 ± 1,8 (SD) (from doctor contact)
Tewhaiti-Smith et al. 2022 [35]	Online survey in New Zealand in 2021 (*n* = 800)	Women with self-reported endometriosis	New Zealand (mean value) 8,7
Mousa et al. 2021 [36]	Unicenter study; retrospective survey (standardized questionnaire) in the United Arab Emirates in 2018 to 2019 (*n* = 518)	Out- and inpatient women, currently diagnosed with endometriosis	UAE (mean value) 11,6 ± 5,6 (SD)
Dmowski et al. 1997 [37]	Unicenter study; retrospective analyiss; questionnaire, interviews, prior medical records in the USA in 1987 to 1995 (*n* = 693)	Consecutive patients treated for endometriosis and chronic pelvic pain or endometriosis ans infertiliy	USA (mean value): 6,4 ± 5,4 (SD) (women with cpp-symptoms^c^); 3,1 ± 2,6 (SD) (women with infertility)

^a^ mean value = arithmetic mean; SD = standard deviation; range = Span of smallest and largest measured value; median = Central value that divides the measured values into two equal halfs

^b^ Italy, Brasil, Argentina, USA, Great Britain, Spain, Belgium, Ireland, China, Nigeria

^c^ Chronic pelvic pain symptoms

According to the results of a multi-center survey across ten countries [30], the delay in endometriosis diagnosis is an average 6.7 years internationally, whereas the time to diagnosis was shortest in China (3.3 years) and longest in Italy (10.7 years) [30]. Studies in other non-European countries revealed that the average (median) diagnostic delay 7 years in Brazil and 11.7 years in the USA [29]. The delays in individual European countries varied between 6.7 years in Norway, 8 to 8.5 (median) years in the United Kingdom, and 10.4 years in Germany and Austria [20, 21, 28, 31]. The mean time from first symptom onset to diagnosis of endometriosis was estimated at 7.8 years in a recent survey in Australia [33], 5.4 years in a similar cross-sectional study in Canada [34], 8.7 years in New Zealand and 11.6 years in the United Arabien Emirates.

Surrey et al. classified the length of diagnostic delay, defined as the mean time from first symptom onset to the date of the first medical insurance claim with an endometriosis diagnosis code (ICD 9/10), as short (≤ 1 year), intermediate (1–3 years) or long (3–5 years) in a study population in the USA (Surrey et al. 2020). However, they limited diagnostic delay to a pre-diagnostic index period of 5 years based on the preliminary results of Soliman et al. [22]. None of the other publications identified in the present review specified any clear cut-off points.

Because of discrepancies between the lengths of pre-diagnostic index periods used to define endometriosis diagnostic delay in the identified studies (Table 1), generally applicable cut-off measures for discrimination between short and long diagnostic delays cannot be derived from the literature. Theoretically, the cut-off point could lie anywhere between 5.4 years and 11.7 years, the minimum and maximum range of pre-diagnostic index periods used in these studies.

Therefore, we used an argumentative approach to cut-off point selection based on our own empirical data, collected from the German population of the EndoCost study. The total diagnostic delay (DDtotal), defined as the mean time (± standard deviation, SD) between symptom onset and confirmed diagnosis of endometriosis, was 7.0 ± 7.3 years (median 5.0 years) for the overall population. The total diagnostic delay period was then divided into a two pre-diagnostic intervals: patient delay (DDpatient), defined as the mean time from the patient first noticing the symptoms of endometriosis to first consulting a medical doctor, and physician delay (DDphysician), defined as the mean time from the patient’s first consultation with a physician to confirmation of the diagnosis. Women in the German population of the EndoCost study had a patient delay (mean ± SD) of 2.8 ± 5.6 years (median 0.0 years) and a mean physician delay of 4.2 ± 5.9 years (median 1.0 years).

The selection of a cut-off point (threshold) should be based on key criteria, such as good statistical and discriminatory power. Theoretically, the cut-off could be set at the arithmetic mean of the total diagnostic delay (DDtotal), which was ≤ 7 years in the EndoCost study. However, it would be unacceptable to classify a period of up to 7 years as “short”, not only from the patient perspective.

The cut-off could also be set at the median of the distribution of diagnostic delay times for the population. The median is a commonly used statistical measure of position [38] which, unlike the arithmetic mean, is not influenced by extreme values. The median diagnostic delay time for the EndoCost study population was 5 years. Women with ≤ 4 years between first symptom onset and diagnosis (DDtotal) were assigned to the shortDD group (*n* = 63), and those with ≥ 5 years were assigned to the longDD group (*n* = 67).

### Study population

A total of 157 women in the German arm of the EndoCost study completed the study questionnaire, corresponding to a response rate of 20%. Twenty-seven of these women were excluded due to missing or unclear diagnostic delay data, leaving a study population of 130 endometriosis patients with the following characteristics:

- Age: 37.9 ± 8.0 years (mean ± SD); range: 19 to 67 years (youngest to oldest)
- Marital status: married: 52%; in a stable relationship: 31%; single, divorced or separated: 17%
- Highest education level: university or technical university degree: 38%; general college entrance qualification: 14%; secondary school leaving certificate (*mittlere Reife*): 42%; lower secondary school leaving certificate (*Hauptschule*): 6%
- Employment status: employed: nearly 80%; in training: 7%; housewife: 5%; *n* = 7 were unable to work due to endometriosis; *n* = 5 were unable to work due to other reasons.
- Personal monthly income: ≤ 500 EUR: 15%; 500 to 1,500 EUR: 47%; 1,500 to 3,000 EUR: 33%; > 3,000 EUR: roughly 5%.

**Fig. 1:**
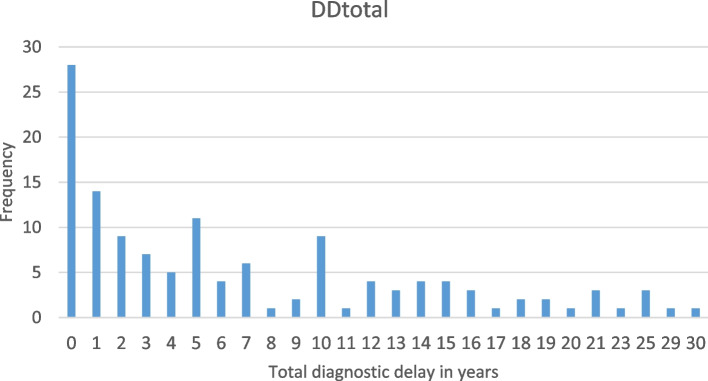
Distribution of the diagnostic delay (Frequencies)

The diagnostic delay time ranges reported by the women who participated in the EndoCost study exhibited a wide range of variation (Fig. 1).

### Comparative analysis: short vs. long diagnostic delay

Our comparative analysis of *n* = 130 women who participated in the German arm of the EndoCost study revealed no significant differences in sociodemographic characteristics between those with a shortDD (≤ 4 years` time between first symptom onset and confirmed diagnosis) versus longDD (≥ 5 years). The results are summarized in Table 2.

**Table 2:** Sociodemographic differences between women with shortDD vs. longDD

Variables	Total-Population		shortDD < 5 years		longDD ≥ 5 years		Mean value diffe-rences	Signifi-cances
*Age at…*	*n*	*Mean values*	*n*	*Mean values*	*n*	*Mean values*		*p-values*
Study entry	130	37,9 (8,0)	63	38,1 (8,3)	67	37,6 (7,8)	0,5	0,724^b^
Onset of symptoms	130	25,6 (11,1)	63	32,3 (9,8)	67	19,3 (8,2)	13,0	≤ 0,001***
First physician consultation	130	28,3 (10,4)	63	32,7 (9,6)	67	24,2 (9,5)	8,5	≤ 0,001***
Confirmed diagnosis	130	32,5 (8,3)	63	33,4 (9,5)	67	31,6 (6,9)	1,8	0,201^b^
*Demographics*								
Size in cm	130	167,8 (8,3)	63	168,9 (7,0)	67	166,7 (6,3)	2,2	0,060^b^
Weight in kg	130	64,9 (11,0)	63	65,6 (10,2)	67	64,2 (11,6)	1,4	0,471^b^
BMI	130	23,0 (3,8)	63	23,0 (3,3)	67	23,1 (4,2)	0,1	0,840^b^
	*N*	*Median*	*N*	*Median*	*N*	*Median*		
Marital status^1^	130	2,0	63	2,0	67	2,0		0,178 ^a^
Highest educational qualification^2^	130	3,0	63	2,0	67	3,0		0,547 ^a^
Monthly net income^3^	130	2,0	62	20	65	2,0		0,878 ^a^

^a^ Mann–Whitney-U-Test, ^b^ Student’s t-test

^1^ Categorical variable with: 1 = single with partner, 2 = married, 3 = single without partner, 4 = divorced/separated, 5 = widowed

^2^ Categorical variable with: 1 = lower secondary school leaving certificate, 2 = secondary school leaving certificate, 3 = general college entrance qualification, 4 = technical university degree, 5 = university degree, 6 = postgraduate, 7 = no degree / certificate

^3^ Categorical variable with: 1 < 500€, 2 = 501–1500€, 3 = 1501–3000€, 4 = 3001–5000€, 5 > 5000€

Overall, compared to women with a shortDD, women with a longDD were not only 13.0 years younger at the age of first symptom onset (19.3 vs. 32.3 years, *p* < 0.001), but also 8.5 years younger (24.2 years old) at the time of first consultation with a physician (*p* < 0.001). However, the difference in age at the time of confirmed diagnosis was no longer significant (women with a longDD were only 1.8 years younger).

There were also significant differences in patient delay (DDpatient), physician delay (DDphysician) and total diagnostic delay (DDtotal) between the groups (*p* < 0.001). Differences in time to diagnosis among women with shortDD vs. longDD are presented in Table 3. DDpatient, defined as the mean time from the patient first noticing symptoms of endometriosis to consulting a medical doctor, was 5.0 years in women with a longDD compared to 0.5 years in those with a shortDD. DDphysician, defined as the mean time from first consultation to confirmed diagnosis, was 7.5 years in women with a longDD compared to only 0.7 years (nearly 7 years shorter) for those with a shortDD. DDtotal was 1.2 years in women with a shortDD compared to 12.4 years in those with a longDD, corresponding to an 11.2-year difference in delay time between the two groups.

**Table 3:** Differences in time to diagnosis among women with shortDD vs. longDD

Variables	Total-Population		shortDD < 5 years		longDD ≥ 5 years		Mean value diffe-rences	Signifi-cances
*Diagnotic delay in years…*	*n*	*Mean values*	*n*	*Mean values*	*n*	*Mean values*		*p-values*
DDwoman	130	2,8 (5,6)	63	0,5 (1,0)	67	5,0 (7,1)	4,5	≤ 0,001
DDphysician	130	4,2 (5,9)	63	0,7 (1,2)	67	7,5 (6,6)	6,8	≤ 0,001
DDtotal	130	7,0 (7,3)	63	1,2 (1,3)	67	12,4 (6,4)	11,2	≤ 0,001
Duration of illness	130	5,3 (5,4)	63	4,7 (5,8)	67	5,9 (5,1)	1,2	0,188
Number of doctor consultations until diagnosis	129	3,0 (2,4)	63	1,9 (1,5)	66	4,1 (2,6)	2,2	≤ 0,001

In Table 4 group differences in endometriosis specific symptoms and visits to general practitioners and specialists are listed. We did not detect any differences neither in pain severity nor in the ASRM-Score (as a classification of the proliferation of endometriosis) between patients with a shortDD versus longDD. However, women in the longDD group consulted significantly more physicians before receiving a confirmed diagnosis of endometriosis.

**Table 4:** Differences in pain severity in women with shortDD vs. longDD

Symptoms and service utilization	Total-Population		shortDD < 5 years		longDD ≥ 5 years		Mean value diffe-rences	Signifi-cances
	*n*	*Mean values*	*n*	*Mean values*	*n*	*Mean values*	*Mean values*	*n*
Average pain intensity	74	4,8 (2,3)	37	4,8 (2,3)	37	4,8 (2,4)	0,0	1,000^a^
Highest pain intensity	75	6,4 (2,5)	37	6,5 (2,5)	38	6,3 (2,6)	0,2	0,671^a^
ASRM-Score	97	2,2 (0,8)	52	2,2 (0,8)	45	2,3 (0,8)	0,1	0,493^a^
Number of physicians consultations until confirmed diagnosis	129	3,0 (2,4)	63	1,9 (1,5)	66	4,1 (2,6)	2,2	≤ 0,001^a^

^a^ Student’s t-test

### Contextual analysis

#### Bivariate correlation analysis

The results of bivariate correlation analysis between the respective dignostic delay and age variables are presented in Table 5.

**Table 5:** Results of bivariate correlation analyses between age variables and diagnostic delay variables

	**Age first symptoms**	**Age first physician consultation**	**Age at diagnosis**
DDtotal	-,695^a^	-,454^a^	-,085
DDphysician	-,433^a^	-,570^a^	-,086
DDwoman	-,423^a^	-,029	-,109

^a^ Significant at the 0.01 level

**Fig. 2:**
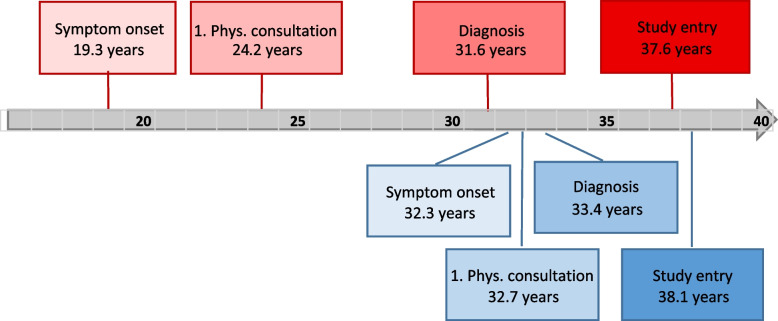
Comparison of the phases of the diagnostic delay of the group with longDD (red) and shortDD (blue)

The analysis revealed a significant negative correlation between age at first symptom onset and all of the diagnostic delay variables (DDpatient, DDphysician, and DDtotal). This correlation was also evident in the further course of the disease as there was also a significantly negative correlation between patient age at first consultation with a physician and a longer diagnostic delay. No correlation between the age at confirmed diagnosis and any of the diagnostic delay variables was detected. This was in agreement with the results of the descriptive analysis, which likewise showed no significant difference in age at diagnosis between the shortDD and longDD group. Figure 2 provides a graphic representation of differences between the two groups.

#### Multiple regression analysis

Regression analysis was performed to determine the nature of association between the various age and diagnostic delay variables. First, we examined the effect of age and the diagnostic delay variables on membership in the shortDD versus longDD group. The following variables were included in the logistic regression analysis: height, weight, ASRM, marital status, educational status, income, age at study entry, age at symptom onset, age at first consultation with a physician, age at confirmed diagnosis, average pain intensity, maximum pain intensity score, and disease duration. Table 6 provides an overview of the results of the regression analysis with the target variable group membership.

**Table 6:** Results of logistic regression analysis for the target variable group membership (shortDD vs. longDD) (*n* = 129)

Variables	Regression coefficient B	Wald-test	Significance	Exp (B)	Confidence interval
Size in cm	-,025	,023	,880	,975	,704 – 1,351
Weight in kg	-,037	,031	,861	,964	,635 – 1,462
BMI	,096	,027	,870	1,100	,350 – 3,460
Educational status	,103	,526	,468	1,109	,839 – 1,465
Marial status	,148	,538	,463	1,159	,781 – 1,719
Personal net income	,097	,155	,694	1,102	,679 – 1,787
Age at study entry	,034	,923	,337	1,035	,965 – 1,110
Age at symptom onset	-,592	18,990	≤ 0,001	,553	,424 -,772
Age first physician consultation	,445	12,068	,001	1,560	1,214 – 2,005
Age at confirmed diagnosis	-,053	2,213	,137	,949	,885 – 1,017
Average pain intensity	,228	,918	,338	1,256	,788 – 2,002
maximum pain intensity score	-,188	,807	,369	,829	,550 – 1,249
Disease duration	-,053	,992	,319	,949	,855 – 1,052
ASRM Score	,209	,367	,545	1,231	,628 – 2,416

None of the demographic or disease-related variables studied had a significant effect on the length of the diagnosis delay. The only variables with a significant effect on group membership (shortDD versus longDD) were age at first symptom onset and age at first consultation with a physician. The negative value of regression coefficient B for age at first symptom onset and Exp B value < 1.0 mean that a young age at symptom onset was associated with a higher risk of a longDD. Moreover, older age at first consultation with a physician was associated with a higher probability of membership in the longDD group.

#### Discriminant analysis

Discriminant analysis was performed to predict group membership in the shortDD versus longDD group based on a multivariate combination of interval variables and normally distributed variables (Shapiro–Wilk test). The canonical correlation coefficient (0.809) exhibited good discrimination between the groups, and the significance level of Wilks’ lambda (*p* < 0.001) demonstrated highly significant differences between the groups. The results of the summary classification table confirm that 90.5% of the originally coded cases were correctly classified. The standardized discriminate function coefficients calculated for each variable (only those > 2) are listed below (Table 7).

**Table 7:** Results of the discriminant analysis (*n* = 129)

Variable	Standardized discriminant coefficients
Age at symptom onset	-1,845
Age at first physician consultation	1,812
Diagnosis delay physician	1,056

The variables with the strongest power to predict membership in the shortDD versus longDD group were age at first symptom onset and age at first consultation with a physician due to endometriosis-associated symptoms, followed by physician delay, defined as the mean time from initial consultation to confirmed diagnosis. Higher age at symptom onset and diagnosis predicted membership in the shortDD group.

Based on the results of these analyses, it can be concluded that that the onset of endometriosis symptoms occurred 13 years earlier in women with a longDD (≥ 5 years) than in those with a shortDD. Consequently, women in the longDD group were roughly 8.5 years younger at the age of first consultation with a physician for endometriosis-related symptoms.

Younger age at first symptom onset was associated with a longer mean time from first symptom onset to first consultation with a physician (DDpatient) and a longer mean time from first consultation to confirmed diagnosis (DDphysician). Moreover, younger age at first symptom onset was associated with an increased risk for a longDD.

## Discussion

### Determination of the cut-off value

A longDD is typical of the clinical management of endometriosis [33, 39]. Potential causes of this delay have been described in the literature [22, 32, 40–42]. What is undisputed is that the affected women perceive the long time from symptom onset to diagnosis as very burdensome, and that the diagnostic delay is associated with high economic burdens [7, 20, 21, 23]. Therefore, having a defined target corridor for diagnosis is as important for physicians as it is for endometriosis patients. Decision limits for defining which diagnostic interval is appropriate have not been determined in studies to date. However, such a cut-off value is needed to be able to identify and a diagnostic delay that is "too long” and to assess its impacts.

The selection of a diagnostic interval of 5 years as the cut-off for differentiating between a shortDD and longDD for endometriosis seems realistic in light of the complexity of the diagnostic process and its good separation power for the study variables. This cut-off value allowed us to divide the study population into two significantly different groups and is in agreement with the average diagnostic delay reported in previous studies [34, 43]. In this respect, calculation of the cut-off limit as the median diagnostic delay time for the study population is based on factually and statistically supported considerations, but a certain degree of arbitrariness remains as a methodological impasse that cannot be overcome. Further statistical analyses of the data (and of qualitative interviews, if necessary) are needed to evaluate the robustness of the selected cut-off value.

### Characteristics of group differences

Group comparison clearly showed that the women in the longDD group were significantly younger (roughly 20 years younger) at the time of first symptom onset than those in the shortDD group (early 30 s). Bivariate correlation analysis of all study variables for the German population of the EndoCost study revealed that the strongest correlation of age at symptom onset was with DDtotal: the younger the age at symptom onset, the longer the patient and physician delay intervals and, thus, the longer the total diagnostic delay. Our correlation, regression, and discriminant analyses showed that age at symptom onset and age at first consultation with a physician significantly influenced the length of diagnostic delay (and, thus, group membership). This is comparable to the findings in other study populations [22, 44].

A gynecological practice study of 653 patients, in which the diagnostic delay for endometriosis was analysed according to the type of dysmenorrhea revealed that the diagnostic delay for women with primary dysmenorrhea (17.6 years on average) was more than twice as long as that for women with secondary dysmenorrhea (8.3 years), and that women with primary dysmenorrhea had first symptoms at a very early age (at 12.7 years), while secondary dysmenorrhea occurred exactly 10 years later (on average among the women surveyed) [45].

Interpretation of the present results allows the following conclusions: Statistically, the two groups did not differ in age, marital status, educational status or income at the time of study enrolment (Table 2). The main difference was in the duration of endometriosis symptoms: by the study enrolment date, women in the longDD group had been experiencing typical endometriosis symptoms for nearly 19 years compared to “only” 6 years for women in the shortDD group. In view of the fact that this is a very young target group, it must be emphasized that the women with a long diagnostic delay of diagnosis were impaired in various stages of life that are important for later life planning. In particular, these patients had no contact with a physician who could have diagnosed endometriosis during their school years, training, and at the start of their career. Against this background, women in Germany are entitled to annual cancer screening by a gynecologist from the age of 20.

Close attention must be paid to the highly significant difference in diagnostic delay between the two groups evaluated in this study. Our analysis of the data revealed a phenomenon in women with a longDD, which has already been discussed in qualitative interviews: one possible explanation is that the young age of the affected women at the onset of endometriosis symptoms means that they lacked the knowledge to differentiate between normal and abnormal menstrual experiences, which could lead them to misjudge the relevance of their symptoms [10, 46, 47]. For pubertal and adolescent girls, the most important and often the only sources of information are friends and family members, who often tend to normalize, play down and trivialize their menstrual irregularities and view them as “bad luck” or “fate”, partly based on their own experiences [10, 39, 48]. In case of a young age at onset of endometriosis symptoms, a young female’s healthcare access is strongly dependent on her parents or guardians and their recognition that the symptoms require medical attention. Finding further valid information sources would probably be too much of a challenge for young females of this age, and an Internet search would probably be unproductive if they are unfamiliar with the term "endometriosis". In this respect, it is not surprising that they tend to adopt the views of the people in their immediate environment and do not question them in later years [49]. In many cases, affected women do not change their minds until further worsening of endometriosis symptoms or infertility problems lead them to seek medical attention. Valuable time is lost until the patient is diagnosed, during which progression of this chronic proliferative disease can occur [50]. The situation is different for affected women with a shortDD. Like other researchers [22, 45], our data suggest that women with a shortDD are older at the time of symptom onset, which enables them to access health information and healthcare providers in an independent and self-determined manner. This is associated with a short time interval from the patient’s first noticing the symptoms of endometriosis to first consulting a physician to receiving the final diagnosis.

The above-mentioned associations between an early onset of symptoms of endometriosis and diagnostic delay, defined as the time from symptom onset to final diagnosis also apply to the (iatrogenic) physician delay. In women with a longDD, the process of establishing the diagnosis was not completed until a long time after the first physician contact. This raises the question of why women who were just under 25 years of age at first physician contact had to wait 7 years for a confirmed diagnosis, whereas those who were over 30 at first physician contact got a confirmed diagnosis within three-quarters of a year. Qualitative studies by Ballard [20] and De Bie/van den Berg [51] provide a possible explanation for this. Their data suggest that due to the normalization of pain that they experienced over the years, younger women may tend to play down their symptoms when talking to a physician, thus increasing the risk that the doctor might not find the symptoms severe enough to consider the possibility of endometriosis. These women are also in a stage of life characterized by a variety of changes, such as the transition from school to vocational training, university, job and new or changing partnerships). The associated changes of residence may be a reason for the well-known phenomenon of doctor hopping, leading to a lack of continuity of healthcare providers which, given the complexity of the differential diagnosis of endometriosis, could further complicate the diagnostic process. In addition to also finding that younger age at symptom onset is associated with a longer diagnostic delay, one study revealed that the diagnostic delay for women whose main complaint was infertility was shorter than that for women whose primary complaint was pelvic pain [29]. Another author suggests that because of fear of stigmatization, women may actively conceal their menstrual irregularities through practices of “menstrual etiquette” [52].

Regarding factors contributing to the delay in diagnosis of endometriosis at the medical level, various authors [10, 20] have surmised, that the attending physicians themselves tend to normalize menstrual pain, forego comprehensive examination procedures such as laparoscopies, and hastily prescribe hormone therapies (mainly oral contraceptives) in an attempt to provide pain relief [20]. The results of a quantitative study conducted by researchers in the Netherlands support this view [51]: These researchers determined, that only 35% of endometriosis patients in their study population had received a physical examination at their first consultation with their primary care physician, and that women who had received a physical examination at first consultation with a physician had a significantly shorter diagnostic delay (5.4 ± 7.1 years) than those who did not. Furthermore, they stressed the importance of proactive collaboration between the primary care physician, gynecologist and patient and emphasized the importance of “considering it" for all parties involved: the only way to minimize the diagnostic delay is if the affected patients take their complaints seriously and if their primary care physicians and gynecologists perform a comprehensive examination [51]. Purely symptomatic treatment with Dienogest without a confirmed diagnosis which is the preferred management approach recommended in the ESHRE guideline [53] has a positive effect on the symptoms of endometriosis, but it sometimes does not significantly slow down the activity of the disease itself. The hormonal suppression of symptoms lulls physicians and patients into a false sense of security. There is a lack of studies to demonstrate the efficacy of hormone therapy in adolescents over a very long period of time (several years), including consideration of compliance at this stage of life. The extent to which the quality of care of endometriosis patients is influenced by other factors, such as poor compensation, time shortages, complacency and cost aversion (patients must pay for the conceptive pill out of pocket) remains to be investigated in future studies. The many unanswered questions about the etiology, diagnosis and treatment of endometriosis attest to the complexity of the disease and pose great challenges to the physicians treating women affected by the disease [54].

### Limitations

This study, based on data from the German arm of the EndoCost study, is subject to various limitations. Firstly, because the patients were recruited through certified endometriosis centers and facilities specializing in fertility treatment, there is a high probability of selection bias in favor of endometriosis patients with more severe disease or with an unfulfilled desire to have children. Secondly, considering that the date of onset of first symptoms of endometriosis was retrospectively determined based on patient self-reports and that a patient’s retrospective self-perception of symptoms as endometriosis is subject to very individual differences, a very detailed clinical history would probably be necessary to confirm the self-reported data. Another source bias is that the study population had a higher level of education than the average educational level of age-matched women [35].

The low response rate of 20% suggests a high level of self-selection bias and, thus, the underrepresentation of certain population groups in the target population. Thus, the patient self-reports may have been collected mainly from a population of endometriosis patients who happened to have active disease and a high disease burden at the time of the study and therefore decided to participate. Furthermore, other patient characteristics, such as a migration background, were not taken into account. Studies with larger sample sizes are required to validate the present results in this respect.

Various statistical analysis methods were used to demonstrate the robustness of the results of the present study. However, the correlation between early age at symptom onset and long diagnostic delay observed here reveals a limitation to the interpretation of the results. Multicollinearity may be a problem if correlations between predictor variables that may affect the interpretation of regression coefficients exist. The correlation analyses did indeed show low to moderate correlations between age at symptom onset / first physician contact and total diagnostic delay / physician delay. However, our tests of multicollinearity with tolerance values of 0.5 to 0.8 and variance influence factor (VIF) values of 1.2 to 1.8 did not reveal any collinearity between the studied variables [55]. The VIF indicates whether a predictor has a strong linear relationship with the other predictor(s). Although there are no hard and fast rules about what value of the VIF should cause concern, Myers [56] suggests that a value of 10 is a good value at which to worry. What’s more, if the average VIF is greater than 1, then multicollinearity may be biasing the regression model [55]. Related to the VIF is the tolerance statistic, which is its reciprocal (1/VIF). As such, values below 0.1 indicate serious problems, although Menard [57] suggests that values below 0.2 are worthy of concern [58].

## Conclusions

We consider the identification of a cut-off value for endometriosis diagnostic delay to be a key finding of the present study based on an analysis of data from the German arm of the Endo-Cost study. According to the results of this analysis, the confirmed diagnosis of endometriosis should be made within less than five years from symptom onset in order to minimize the risk of an unfavorable course of the disease as shown for example by Surrey et al. [23].

Because there are so many very different reasons for the delay in diagnosis of endometriosis, the main factors contributing to this delay must first be determined. Our analysis of data from the EndoCost study confirmed the findings of other studies suggesting that an early onset of endometriosis must be regarded as a significant risk factor for a long diagnostic delay. This highlights the importance of educating adolescent girls and young women as well as people in their environment about endometriosis as a first important approach to improving endometriosis care. However, the continuing lack of knowledge and awareness of this topic in society remains a barrier to access to the target group of adolescents and young women [10]. The evidence suggests an urgent need to develop target group-specific strategies for informing target groups about normal and pathological menstrual symptoms [59]. The aim of information and education campaigns in the school and new media context should be to get adolescent girls and young women to consult a physician earlier and to give the doctor an authentic description of their symptoms, particularly menstrual cycle-related pelvic pain.

The physicians who treat adolescent girls and young women (general practitioners, pediatricians, gynecologists) are another important target group. They should be (even) better educated and, in particular, have even higher awareness of the possibility that endometriosis can occur even in women well under the age of 30 years to ensure that differential diagnostic methods will be employed at an earlier stage. The implementation of a structured concept for the creation and certification of endometriosis centers in Germany and other European countries is important for improving the endometriosis-specific know-how of healthcare professionals [60]. Further action is needed. Like other endometriosis researchers [42, 53, 61–64], we emphatically stress the need for greater awareness of the disease, better education and more intensive cooperation not only at the level of the patient, healthcare provider, science and health policy, but also at the level of society as a whole.

## Data Availability

The datasets generated and/or analyzed during the current study are only available from the corresponding author on reasonable request due to patient privacy considerations and the german data protection law.

## Abbreviations

ASRM: American Society of Reproductive Medicine
AWMF: Arbeitsgemeinschaft der Wissenschaftlichen Medizinischen Fachgesellschaften e.V
BMI: Body mass index
DDpatient: The patient delay interval, defined as the mean time from first symptom onset to first consultation with a physician
DDphysician: The physician delay interval, defined as the mean time from first consultation to confirmed diagnosis
DDtotal: The total diagnostic delay, defined as the mean time from first symptom onset to confirmed diagnosis
ESHRE: European Society of Human Reproduction and Embryology
IBM SPSS: Statistics software
ICD: International Statistical Classification of Diseases and Related Health Problems
SD: Standard deviation
ShortDD: Short diagnostic delay
longDD: Long diagnostic delay
SPSS: Statistical Package für Social Sciences. VIF: variance influence factor
WERF: World Endometriosis Research Foundation
